# Testing the validity of the Norwegian translation of the modified weight bias internalization scale

**DOI:** 10.1186/s40337-024-01067-z

**Published:** 2024-08-15

**Authors:** Tiffany Lussier, Jon Harald Quindao Tangen, Trine Tetlie Eik-Nes, Håvard R. Karlsen, Kjersti Hognes Berg, Charlotte Fiskum

**Affiliations:** 1 Department of Psychology, Norwegian University of Science and Technology, Trondheim, Norway; 2 Department of Neuromedicine and Movement Science, Norwegian University of Science and Technology, Trondheim, Norway; 3 Stjørdal Community Mental Health Centre, Levanger Hospital, Nord-Trøndelag Hospital Trust, Stjørdal, Norway

**Keywords:** Weight bias internalization, Modified weight bias internalization scale (WBIS-M), Weight-related competence, Weight stigma, Internalized stigma

## Abstract

**Background:**

Individuals with higher weight (overweight or obesity) may experience social stigma due to their weight. Weight stigma can be internalized with adverse health effects. Internalized weight stigma is relevant across different weight categories, but no validated weight-neutral measure of internalized weight bias currently exists in Norway. The current study aimed to examine the validity of a Norwegian translation of the Modified Weight Bias Internalization Scale.

**Methods:**

A Norwegian translation of the Modified Weight Bias Internalization Scale (WBIS-M) was administered in an adult Norwegian sample (*N* = 315, of which 251 women) ranging from self-reported “very underweight” to “very overweight”.

**Results:**

A confirmatory factor analysis was conducted on 11 of the original 11 items in the WBIS-M. Based on previous factor analyses with this scale, we expected a one-factor model. One of the items related to competence showed poor model fit, and concern was raised around possible item ambiguity partway through the study. Two versions of this item were therefore tested, neither of which yielded an acceptable fit. After exclusion of this item, the results showed high loadings for the remaining 10 items on one factor with a high internal consistency (α = 0.94). Convergent validity was approached by looking at the relationship between answers on the WBIS-M, self-perceived weight, and items on overall health and psychological/emotional state.

**Conclusion:**

The 10-item Norwegian version of the WBIS-M shows sound psychometric properties and can be used to measure internalized weight bias in a weight-neutral fashion in a Norwegian-speaking population. Internalized weight bias was correlated with psychological/emotional state and overall health, with those reporting more internalized weight bias also reporting that they felt worse. This relationship was stronger for women than men in our sample and was partially dependent on weight. The women also showed higher internalized weight bias than the men. Future studies should include more male participants and explore alternative versions of the missing item related to competence.

## Abstract (plain language summary)

Individuals with higher weight may experience social stigma due to their weight. This can include encountering beliefs that people of higher weight lack willpower or are not as smart or valuable as others. Stigma related to weight can then be internalized, leading to *weight bias internalization*, which is when people start to believe in weight-related stigma about themselves. A Norwegian translation of the modified weight bias internalization scale (WBIS-M) was answered by a Norwegian sample. A confirmatory factor analysis was conducted to determine whether the scale had a single-factor structure. The results showed that 10 of the original 11 items fit well, but one item related to the experience of competence should be removed. Once this item was removed, the scale had good statistical properties, indicating that internalized weight bias can be measured by the 10 items of the Norwegian WBIS-M. Internalized weight bias was related to how well people were feeling psychologically and health-wise, with those reporting more internalized weight bias also reporting that they felt worse. This relationship was stronger for women than men in our sample. Future studies should include more male participants and explore further versions of the competence item.

## Background

Individuals with overweight or obesity can experience social stigma because of their weight in multiple settings. Weight stigma usually involves people talking and acting in a way that makes those who are affected feel that they are less moral, lack willpower, or are not as smart or valuable as others [1].

Weight stigma is prevalent internationally [2, 3], and discrimination against individuals in higher weight populations has been documented in professional and medical settings in Norway [4, 5]. Weight stigma can also be internalized, leading to weight-bias internalization (WBI), which occurs when affected individuals become aware of the negative weight-related stereotypes attributed to them, agree with them, and subsequently apply these stereotypes to themselves. The WBI construct focuses specifically on weight-related self-evaluations and is distinct from body image, which applies more generally to one’s evaluations of and investment in one’s body and its characteristics [6]. While the negative health outcomes of social stigma toward people in higher weight populations are well documented [1], adverse outcomes associated with self-directed stigma require further study. To date, associations between WBI and reduced health and worse outcomes have been found, including studies showing that depression, anxiety, and disordered eating are associated with higher WBI [7–9]. Studies also show negative associations between WBI and physical health, but this has not been as extensively researched [9]. Further study is still warranted, necessitating ways to measure WBI across populations.

The 11-item Weight Bias Internalization Scale (WBIS) was originally developed by Durso and Latner [6] to assess internalized weight bias in populations with self-reported overweight, and its items make explicit references to respondents’ higher weight. Previous investigations have shown that the WBIS can be used both with regard to participants’ actual BMI status and self-perceived weight status, with similar associations [10].

The WBIS has shown some inconsistency in psychometric properties in regard to factor structure and item fit [9], and some authors have found it necessary to exclude the first item, which refers to the perception of competence, to achieve good model fit [10, 11]. Another concern related to WBIS has been its lack of suitability outside of populations with self-perceived or actual overweight, as many items contain the wording “as an overweight person…”.

The modified version of the WBIS (WBIS-M) developed by Pearl and Puhl [12] does not refer to specific weight categories and can therefore be used to measure WBI in individuals across weight categories, increasing usability both clinically and in research. The initial validation of the 11-item version of the WBIS-M showed satisfactory properties [12]. However, further examination of the psychometric properties of the WBIS-M is still warranted, particularly related to item 1, which has previously exhibited psychometric challenges in WBIS. Furthermore, there is a need for additional research on the relationship between WBI and overall health [9], as well as on gender differences related to WBI. Men have previously been observed to exhibit weight stigma internalization when they are both underweight and overweight and the BMI threshold to experience WBI may be higher than in women [13–15]. Finally, with 26% of Norwegian women and 35% of Norwegian men qualifying as overweight (BMI equal to or exceeding 27) [16], the study of internalization of weight bias is also relevant in a Norwegian context. Currently, there is no validated weight-neutral scale measuring WBI in Norwegian, and the addition of a validated scale is important for research, mental health practice, and prevention and treatment of weight-related health problems in Norway, as well as our understanding of WBI across different cultures.

The present article aims to investigate the psychometric properties of the Norwegian translation of the WBIS-M. The study also investigated gender differences in WBI using the Norwegian version of the WBIS-M, along with correlations between WBI and self-perceived overall health, psychological well-being, and self-perceived weight status in both men and women.

## Methods

### Participants

A total of 315 participants (men, *n* = 63; women, *n* = 251; n.a., *n* = 1) were recruited among employees from two Norwegian municipalities (one rural and one urban), through social media, and the Norwegian interest organization for people with overweight. As the topic of weight stigma may draw the attention of women more easily than men, men were encouraged to participate in the social media posts. The recruitment called for “generally healthy participants” above the age of 16 who understood Norwegian well. No steps were taken to ascertain health or language status before participants responded to the questionnaire, but all the information was given in Norwegian. No compensation was offered for participation. No subjects under 18 responded.

### Procedure

Respondents completed an anonymous questionnaire on the secure survey service “Nettskjema” from the University in Oslo. Respondents had to give explicit consent to participate before responding to the survey. An informational form describing all rights was provided electronically. In addition, a list of relevant helplines was provided along with the project leader’s contact information.

### Measures

#### The Norwegian version of the WBIS-M

Participants completed a Norwegian translation of the WBIS-M based on the 11-item version [12]. The English WBIS-M was translated into Norwegian by two bilingual researchers and back-translated by a third bilingual translator independently. All translators had experience with working with internalized weight stigma clinically. Discrepancies were settled through discussion between the two first translators, focusing on staying as close to the perceived meaning in the original items as possible. Whether items and the whole of the translated scale had face and construct validity was part of the discussions. Extra attention was given to the first item in the WBIS-M due to previous psychometric issues [10, 11]. A translation that was as close to the original wording as possible was initially chosen, with the back-translation coming back identical to the original. An inspection of the distribution of answers to the first item midway through the data collection gave rise to concerns that the item was hard to understand and answer. Specifically, a visual inspection of the distribution indicated a bimodal distribution of answers that could mean the item was ambiguous in terms of whether responses on either end of the agree/disagree-continuum indicated the presence of WBI or not. This was the only item showing this distribution. Preliminary analysis of factor loadings and internal validity (Cronbach’s alpha) also indicated low values for item 1. A further discussion on the wording between the translators revealed concerns around the item’s wording, particularly regarding response style and whether disagreement or agreement indicated WBI or something else. Therefore, the first item ‘‘Because of my weight, I feel that I am just as competent as anyone’’ was re-examined, and a new version using a phrasing directly back-translated as “In spite of my weight, I feel that I am as competent as others” was added to the survey to see if this phrasing improved the item, as well as the ease of interpretation of the response (Table 1). A total of 315 participants responded to the first version, competent1, while 138 completed both versions competent1 and competent2. After data collection was complete, statistical analyses were conducted to try to elucidate if either item version 1 or 2 yielded a better model fit with neither item demonstrating good fit. See the results section for further details.

**Table 1:** Translations of the original English WBIS-M items into Norwegian and their corresponding item names

English original	Norwegian translation	Item name
1. Because of my weight, I feel that I am just as competent as anyone.	På grunn av vekten min føler jeg at jeg er like kompetent som andre	competent1
	Til tross for vekten min føler jeg at jeg er like kompetent som de fleste	competent2
2. I am less attractive than most other people because of my weight.	På grunn av vekten min er jeg mindre attraktiv enn de fleste andre	less_attractive
3. I feel anxious about my weight because of what people might think of me.	Jeg bekymrer meg for vekten min på grunn av hva folk tenker om meg	people_think
4. I wish I could drastically change my weight.	Jeg skulle ønske jeg kunne endret vekten min drastisk	change_weight
5. Whenever I think a lot about my weight, I feel depressed.	Når jeg tenker mye på vekten min, føler jeg meg deprimert	depressed
6. I hate myself for my weight.	Jeg hater meg selv på grunn av min vekt	hate
7. My weight is a major way that I judge my value as a person.	Vekten min har stor påvirkning på hvordan jeg vurderer min verdi som person	my_value
8. I don’t feel that I deserve to have a really fulfilling social life, because of my weight.	Jeg syns ikke at jeg fortjener å ha et tilfredsstillende sosialt liv på grunn av vekten min	social_life
9. I am OK with being the weight that I am.	Jeg er OK med den vekten jeg har	ok_weight
10. Because of my weight, I don’t feel like my true self.	På grunn av vekten min føler jeg meg ikke som mitt “sanne jeg”	true_self
11. Because of my weight, I don’t understand how anyone attractive would want to date me.	På grunn av vekten min tror jeg ikke noen som er attraktive ville ønske å date meg	date

Note: English wording is a reproduction of the original WBIS-M by Pearl and Puhl, 2014

#### Demographic information

Demographic information about the participants, such as gender, age, highest attained level of education, and self-perceived weight, was gathered. Participants’ age was coded on an interval scale ranging from 16 to 18 years to ≥ 66 years. The choice to use age-brackets was made after the Norwegian Agency for Shared Services in Education and Research (SIKT) recommended using brackets due to privacy concerns. The first two intervals were distributed in blocks of two years (16–18 and 18–20) due to possible needs to filter out under-18-year-olds, while the age categories between 21 and 66 were coded in five-year blocks (e.g., 21–25, 26–30, etc.). Ages 66 + were coded as one category. No participants were under 18, and only 5 participants were over the age of 66. The distribution of age categories approximated a normal distribution: 19–20 (0.6%), 21–25 (9.5%), 26–30 (13.7%), 31–35 (11.7%), 36–40 (11.4%), 41–45 (19.4%), 46–50 (12.4%), 51–55 (11.1%), 56–60 (4.4%), 61–65 (4.1%) and 66 and over (1.6%). Educational attainment was categorized on a 3-point scale ranging from high school to vocational school or less (*n* = 43), bachelor’s degree (*n* = 100) or master’s degree or higher (*n* = 172).

Participants were asked to rate their weight status on a 7-point Likert scale ranging from 1 – Very underweight, 4 – Normal weight, to 7 – Very overweight. Thirteen (4.1%) of the respondents described their weight as being underweight or slightly underweight (points 2 to 3), 155 described themselves as normal weight (point 4, 49.2%), and 147 (46.7%) respondents reported themselves to be slightly overweight (92), overweight (40) or very overweight (15 respondents). Height and weight were not collected and therefore BMI values were not calculated. We can therefore not ascertain the relationship between participants’ perceived and actual weight status.

#### Health status

Finally, participants were asked to report their health status using the Dartmouth Coop Functional Health Assessment/World Organization of National Colleges, Academies and Academic Association of General Practitioners (COOP/WONCA) form [17]. The COOP/WONCA scale has been validated in Norwegian settings [18, 19] and assesses perception of overall health and psychological and physical state using a 5-point scale wherein lower scores are indicative of greater health. For this study, the measures of *overall health* and *psychological/emotional state* were used.

### Statistical analyses

To validate the structure of the Norwegian version of the WBIS-M, a confirmatory factor analysis (CFA) was conducted using R version 3.4.0 with the lavaan package (v.0.6.16).

Differences between overweight and non-overweight participants, as well as gender differences were investigated using Welch’s t-test in IBM SPSS version 29. Effect sizes were calculated using Cohen’s d with effect sizes below 0.2 being interpreted as small, those between 0.2 and 0.5 being moderate, sizes between 0.5 and 0.8 were considered large, and above 0.8 very large [20]. Correlations between variables were investigated using Pearson’s and Spearman’s rank correlation coefficient in SPSS, depending on distribution of the variables. In line with studies of typical effect sizes in psychological research [21, 22], correlation coefficients of 0.10 and below were considered weak, coefficients of 0.20 – 0.30 were considered moderate, and coefficients above 0.30 were considered large. Due to previously observed associations between WBI and negative mental health outcomes as well as reduced physical health, we expected WBI to correlate negatively with these measures, and positively with self-perceived weight.

For the factor analysis, an initial model in which all the items loaded on a single factor was tested. This model was chosen because it is the identified factor structure from the original scale [6]. In the presence of poor fit, localized areas of strain were identified by inspecting modification indices. If constraints could be justified and introduced based on sound theory or clinical experience, a new model fit was calculated for this model. This continued until a model remained with acceptable fit indices. For all models, the maximum likelihood approach was used to create parameter estimates. To assess model fit, we computed the comparative fit index (CFI), the Tucker‒Lewis index (TLI), the root mean square error of approximation (RMSEA) and the standardized root mean square residual (SRMR). Following guidelines from Hu and Bentler [23], acceptable model fit was defined by these criteria: CFI and TLI at or above 0.95, RMSEA at or below 0.06, SRMR at or below 0.08.

## Results

### Factor analysis

The initial model contained 12 items of the WBIS-M, as the first two items were variants of the competence item with different phrasings, and their error covariances were allowed to correlate (Table 2).

**Table 2:** Factor loadings for model 1

Item	Standard loading	SE	z	*p*	CI.lower	CI.upper
competent1	0.08	0.09	0.91	0.363	-0.09	0.25
competent2	0.27	0.08	3.31	0.001	0.11	0.43
less_attractive	0.83	0.03	28.52	< 0.001	0.78	0.89
people_think	0.74	0.04	18.27	< 0.001	0.66	0.82
change_weight	0.82	0.03	26.18	< 0.001	0.76	0.88
depressed	0.78	0.04	21.87	< 0.001	0.71	0.85
hate	0.76	0.04	19.35	< 0.001	0.68	0.83
my_value	0.6	0.06	10.62	< 0.001	0.49	0.71
social_life	0.56	0.06	9.26	< 0.001	0.44	0.68
ok_weight	0.86	0.03	33.04	< 0.001	0.81	0.91
true_self	0.84	0.03	30.3	< 0.001	0.79	0.9
date	0.77	0.04	0.91	< 0.001	0.7	0.84

Note. The results have been rounded to the second decimal point

The factor loadings were above the recommended cutoff of 0.4 for all items apart from the two competence items.

The model fit indices for the initial model were generally outside the recommended values. The CFI was 0.88, while the TLI was 0.86, indicating unacceptable fit. The RMSEA was 0.13, indicating unacceptable fit. Finally, the SRMR was 0.07, indicating acceptable fit.

Due to the low factor loadings of both translations of the competence items, they were removed from the model. A subsequent model was run with the remaining 10 items of the WBIS (Table 3). All factor loadings were above the recommended cutoff of 0.4 after removal of the competence items.

**Table 3:** Factor loadings for model 2

Item	Standard loading	SE	z	*p*	CI.lower	CI.upper
less_attractive	0.85	0.02	48.49	< 0.001	0.82	0.88
people_think	0.79	0.02	34.56	< 0.001	0.75	0.84
change_weight	0.85	0.02	48.73	< 0.001	0.82	0.88
depressed	0.84	0.02	45.39	< 0.001	0.8	0.88
hate	0.77	0.02	31.07	< 0.001	0.72	0.82
my_value	0.6	0.04	16.05	< 0.001	0.53	0.68
social_life	0.58	0.04	14.95	< 0.001	0.51	0.66
ok_weight	0.84	0.02	44.52	< 0.001	0.8	0.87
true_self	0.82	0.02	40.16	< 0.001	0.78	0.86
date	0.82	0.02	40.98	< 0.001	0.78	0.86

Note. The results have been rounded to the second decimal point

The model fit indices improved, but some indices were still outside of the recommended values. The CFI was 0.94, while the TLI was 0.92, indicating unacceptable fit. The RMSEA was 0.12, indicating unacceptable fit. Finally, the SRMR was 0.05, indicating acceptable fit.

Inspecting the modification indices indicated that fit could be improved by allowing the error of the *hate* item to correlate with the errors of *social_life* and *my_value*. It seemed clinically reasonable to assume that self-hate based on weight could be associated with feelings of lower value and being undeserving of a good social life, so this modification was accepted. A third model including these correlations was run. The factor loadings were similar to Model 2, all being above the recommended cutoff of 0.4 (Table 4).

**Table 4:** Factor loadings for model 3

Item	Standard loading	SE	z	*p*	CI.lower	CI.upper
less_attractive	0.85	0.02	49	< 0.001	0.82	0.89
people_think	0.79	0.02	34.4	< 0.001	0.75	0.84
change_weight	0.86	0.02	50.46	< 0.001	0.82	0.89
depressed	0.83	0.02	42.66	< 0.001	0.79	0.87
hate	0.74	0.03	27.04	< 0.001	0.69	0.79
my_value	0.59	0.04	15.42	< 0.001	0.52	0.67
social_life	0.56	0.04	13.91	< 0.001	0.48	0.64
ok_weight	0.84	0.02	46.07	< 0.001	0.81	0.88
true_self	0.82	0.02	41.11	< 0.001	0.78	0.86
date	0.82	0.02	40.46	< 0.001	0.78	0.86

Note. The results have been rounded to the second decimal point

For this model, the CFI was 0.96, while the TLI was 0.95, indicating acceptable fit. The RMSEA was 0.09, while the SRMR was 0.04, also indicating acceptable fit.

A correlation table of all items is shown in Table 5. Of the 315 respondents, 177 did not answer *competent_2* because it was introduced partway through the data collection phase. There were no missing values on any of the other scales.

**Table 5:** Descriptives and Pearson’s correlation coefficient of all items

Items	1	2	3	4	5	6	7	8	9	10	11	12
1. competent1	1											
2. competent2	0.24	1										
3. less_attractive	0.2	0.26	1									
4. people_think	0.13	0.21	0.7	1								
5. change_weight	0.11	0.11	0.74	0.64	1							
6. depressed	0.08	0.11	0.68	0.72	0.7	1						
7. hate	0.16	0.31	0.64	0.59	0.62	0.7	1					
8. my_value	0.06	0.29	0.46	0.54	0.45	0.53	0.57	1				
9. social_life	0.18	0.39	0.5	0.44	0.45	0.43	0.61	0.49	1			
10. ok_weight	0.1	0.16	0.7	0.64	0.76	0.72	0.59	0.46	0.43	1		
11. true_self	0.09	0.24	0.68	0.63	0.72	0.68	0.59	0.49	0.44	0.75	1	
12. date	0.13	0.25	0.74	0.65	0.71	0.66	0.65	0.48	0.53	0.65	0.66	1
Mean	2.76	1.56	2.26	2.44	2.52	2.33	1.03	2.22	0.71	2.48	2.05	1.89
SD	1.9	1.62	1.94	1.9	2.08	2.01	1.57	1.88	1.29	1.96	2	1.87

Cronbach’s alpha for the 10-item scale was 0.94, indicating good internal consistency.

### Analyses of differences between non-overweight and overweight respondents

A Welch’s t-test showed that non-overweight respondents reported a significantly lower level of WBI than did overweight respondents, with a very large effect (*d* = -1.39) (Table 6). Conversely, non-overweight respondents reported a significantly higher level of overall health than did overweight respondents, with a large effect size (*d* = 0.73). No significant differences were found in psychological/emotional state between these two groups.

**Table 6:** Means and SD for non-overweight and overweight participants and independent samples Welch’s t-tests comparisons

Variable	Non-overweight	Overweight	t	*p*	Cohen’s d	95% CI
WBI ^a^	1.2 (1.13)	2.9(1.33)	147.26	< 0.001	-1.39	[-1.63, -1.14]
Overall health	3.95 (0.81)	3.34 (0.84)	42.07	< 0.001	0.73	[0.5, 0.96]
Psychological state	3.62 (1.07)	3.55 (1.09)	0.31	0.578	0.06	[-0.16, 0.28]

Note. N for non-overweight = 168, N for overweight = 294 based on self-reported weight categories where those selecting 1–3 (underweight) and 4 (normal weight) were classified as non-overweight and those selecting 5–7 were categorized as overweight

For general health and psychological state, values were reversed such that higher values equate to greater health

a: WBI equals mean score across all items included in the 10-item version of WBIS-M Norwegian version

### Analyses of gender differences and correlations

A Welch’s t-test showed that women exhibited a significantly higher level of WBI than did men, with a moderate effect size (*d* = 0.36) (Table 7). No differences were observed between genders in self-perceived weight, psychological state, or overall health.

**Table 7:** Means and SD for men and women and independent samples Welch’s t-test comparisons

Variable	Women	Men	t	df	*p*	Cohen’s d	SE Cohen’s d
Perceived weight ^a^	4.65 (0.94)	4.62 (0.92)	0.263	96.486	0.793	0.037	0.141
WBI ^b^	1.99 (1.49)	1.57 (1.46)	2.536	97.176	0.013	0.355	0.142
Overall health	3.66 (0.88)	3.75 (0.92)	− 0.819	92.220	0.415	− 0.117	0.141
Psychological state	3.59 (1.08)	3.63 (1.05)	− 0.410	97.943	0.683	− 0.057	0.141

Note. For general health and psychological state, values were reversed such that higher values equate to greater health

a: Self-reported Weight categories: 1–3 represented underweight, 4 normal weight and 5–7 overweight.

b: WBI equals mean score across all items included in the 10-item version of WBIS-M Norwegian version

For both genders, higher WBI was significantly correlated with higher self-perceived weight, higher levels of psychological distress and poorer overall health (Table 8). We chose to also run the analysis to control for self-perceived weight to see if higher self-perceived weight canceled out the effect of WBI (Table 9). When controlling for self-perceived weight, the negative correlation between WBI and psychological/emotional state remained significant for men, but not the correlation between overall health and WBI. For women, WBI was strongly negatively correlated with both psychological/emotional state and overall health, even when controlling for self-perceived weight (Table 9).

**Table 8:** Correlation matrix of health and weight variables for men and women separately

Variable	1	2	3	4
1. Perceived weight	1	[0.61***]	[− 0.46***]	[− 0.24]
2. WBI	0.6***	1	[− 0.42***]	[− 0.35**]
3. Overall health	− 0.34***	− 0.57***	1	[0.57***]
4. Psychological state	− 0.02	− 0.4***	0.56***	1

Note. N for women = 251, N for men = 63. Spearman’s correlation coefficient with pairwise deletion was used. Correlations for women are shown in the bottom left quadrant, while correlations for men are shown in the top right and set in brackets. For overall health and psychological state, values were reversed such that high values are indicative of greater health

* *p* < .05, ** *p* < .01, *** *p* < .001

**Table 9:** Spearman’s partial correlations controlling for perceived weight in males and females separately

Variable	1	2	3
1. WBI	1	[− 0.27*]	[− 0.20]
2. Psychological state	− 0.48***	1	[0.53***]
3. Overall health	− 0.48***	0.59***	1

Note. N for women = 251, N for men = 63. Spearman’s correlation coefficient with pairwise deletion was used. Correlations for women are shown in the bottom left quadrant, while correlations for men are shown in the top right and set in brackets. For overall health and psychological state, values were reversed such that high values are indicative of greater health

* *p* < .05, ** *p* < .01, *** *p* < .001

## Discussion

This study analyzed a sample of Norwegian speaking participants over 18 years of age on their responses to the Norwegian version of the WBIS-M [12], a weight-neutral version of the WBIS [6]. When excluding two proposed versions of the competence item, the remaining 10 items had high factor loadings and good model fit, confirming a one-factor solution, as demonstrated in previous studies [6, 10–12]. Therefore, the 10-item version of the Norwegian WBIS-M has satisfactory psychometric properties, and we suggest that it can be used to measure internalized weight bias in Norwegian samples in a weight-neutral fashion. Using a weight-neutral scale such as the WBIS-M over the WBIS has the advantage of being inclusive of all weight categories, improving usability in studies across weight classes, or with individuals who have changed weight status, for instance, due to bariatric surgery, who may present as “normal” or underweight but still experience WBI [12]. Furthermore, using terms such as “overweight” in a sample consisting of only those who self-describe themselves as overweight runs the risk of biasing responses toward greater reported internalized stigma due to priming of negative stereotypes, which the use of this 10-item version of the WBIS-M could minimize. An analysis of the descriptive data showed that most of the sample in the present study identified as normal or overweight. We also found that the degree of internalized weight bias in our Norwegian sample was lower for both genders than in the original validation of the WBIS-M [12], but participants who self-perceived as heavier reported greater levels of WBI and lower overall health.

Our results showing better fit with a 10-item version are in line with previous investigations of the psychometric properties of the WBIS that have indicated that the first item related to competence can be challenging [10, 11]. Initial inspection of answers to the first item halfway through our study indicated that this might also be the case in the Norwegian version of the WBIS-M. Specifically, it became unclear whether agreement with the item indicated presence of WBI or not, due to a distribution of answers indicating possible ambiguity, along with low model fit. To address these concerns, the wording of the item was further discussed amongst the clinician-translators, and two versions of the competence item were tested. To our knowledge, this represents the first attempt to see if different phrasings of this item could improve the fit and clarity of the item that has been published. The results showed that neither version showed satisfactory psychometric validity in the CFA and hence did not contribute to a good model fit. Our work indicates that this item may be particularly hard to convey as weight-neutral – at least in Norwegian. The fact that it is one of only two positively phrased items in the scale could also possibly affect how participants interpret the item and respond to it.

There may also be weight-dependent differences in experience with stigma that may affect the interpretation of this item that our sample did not allow us to explore further. While both underweight and overweight individuals can experience weight stigma, the *types* of stereotypes directed toward these two weight groups differ [24], which in turn can affect the nature of internalized weight bias in different weight categories. For example, it may be likely that individuals categorized as overweight would be more likely to self-stigmatize relative to their competence than those who are normal or underweight, as those categorized as overweight can frequently experience being labeled as lazy or lacking in willpower in the public discourse [1]. Therefore, an investigation of both versions of the competence item in a larger sample with higher proportions of individuals with under- and overweight might yield different results. There may also be cultural concerns in Norway related to the relationship between weight and competence that have not yet been investigated. As mentioned, our sample showed lower WBI than the sample in the original publication on the WBIS-M [12], which could perhaps also affect the tendency to internalize WBI related to competence. Interestingly, the advent of social media may also mean that sub-cultural differences may arise more quickly than before, particularly in some demographic groups such as the young [25], meaning that cultural influences may be less obvious to pin down than before the social media age. Given some previous issues with this competence-related item when measuring WBI [10, 11], it may also be inherently challenging to operationalize the relationship between weight and competence regardless of the sample, culture, and language. Therefore, further work may be needed to include competence in this scale in a reliable and valid way, at least in a Norwegian context. This could include doing qualitative exploration using a focus group on the underlying concept of WBI related to competence, suggesting new ways of operationalizing, and rephrasing this concept in a weight-neutral way. For instance, it may be worthwhile to use phrasing which does not emphasize a comparative aspect with others, such as “*My weight affects how competent I feel*”. Such a non-comparative phrasing could perhaps also be beneficial for other language versions of the WBIS-M.

An investigation of gender differences in our material showed a significantly higher degree of internalized weight bias (WBI) among women compared to men. This coincides with other results wherein women experienced more stigma the greater their weight and are more likely to internalize this stigma [9, 26]. Neither self-perceived weight, psychological/emotional state, nor overall health differed among genders in our study, indicating that the higher WBI in the women was likely not a result of differences in these variables. For both genders, higher WBI correlated with higher self-perceived weight, lower overall health, and a more negative psychological state, in the expected directions. In women, higher WBI was also related to both a more negative psychological/emotional state and lower overall health when controlling for self-perceived weight. For men, this was only the case for WBI and psychological state when controlling for self-perceived weight. These results indicate that there is a more robust relationship between WBI and health in women than men, perhaps related to more widespread discrimination against women of higher weight in some settings [13]. However, greater WBI was related to greater psychological distress for both genders, independent of weight, and WBI should therefore be addressed regardless of gender, particularly in clinical settings.

### Limitations and future research

This study has some limitations that can be addressed in future research. First, the sample size was too small for further analyses that could have shed more light on WBI across different weight categories, particularly because we did not have many underweight participants. Second, the study did not include objective measurements of BMI. However, this was a deliberate choice founded in feedback from user organizations and clinical work in the research group where we have found that the practice of BMI-measurements can be considered stigmatizing and/or triggering for some individuals, particularly with eating disorders [27]. Moreover, previous investigations on WBI have shown that self-perceived weight is an acceptable and efficient measure when investigating WBI and can be used as an alternative to objective BMI [10]. We do not expect our results would have changed significantly with the use of BMI instead of self-perceived weight, although perhaps an over-estimation of self-perceived weight might be related to more WBI than actual BMI for some respondents.

A further limitation is the low number of men (*n* = 63) compared to women included in the sample despite the efforts that were undertaken to recruit more men. We had too few men to be able to perform a valid test of measurement invariance among the gender groups, so we cannot rule out that the scale functions differently for men and women. Future studies ought to verify measurement invariance in this Norwegian translation of the WBIS-M to give more confidence in examinations of gender differences. However, inspection of the correlations between WBI and general health, psychological/emotional state, and self-perceived weight in our sample were similar and in expected directions in both genders, strengthening the assumption that the scale measured the same construct in both genders. The relative lack of men could reflect a weakness in the recruitment strategy. It could also indicate that internalized weight bias is perceived as more relevant to women than men. In line with this, we found that the women in our sample showed more WBI than the men. Again, a larger sample would have allowed us to explore possible nuances of this result. For instance, while not observed in our analyses, other studies have shown that men can show more U-shaped tendencies in internalized weight stigma, wherein they experience it most when underweight or overweight [13, 14]. They may also have a higher BMI-threshold before they perceive themselves as overweight [15]. Therefore, as women were overrepresented in our sample, the validity of the Norwegian WBIS-M scale may be more firmly established using samples with a more equal distribution of genders. Studying a more diverse sample in terms of gender identity might also nuance the results further. We had only 1 respondent indicate a gender identity apart from male/female, which did not allow for further exploration, but future studies could aim for a more diverse recruitment strategy in terms of gender.

Furthermore, the study could have incorporated more measures of WBI for a better exploration of convergent validity. However, there is currently a lack of validated measures of WBI in Norwegian, so we chose to approach the question of convergent and external validity in terms of the relationship to measures known to be related to WBI, such as health status, psychological/emotional state, and self-perceived weight. The presence of face and construct validity was also an important part of the discussions during the translation process, which was conducted by a clinical team with long experience working with WBI in patients with higher weight and eating disorders. Future studies should look at adaptation of other measures of WBI into Norwegian, to compare these with the Norwegian translation of the WBIS-M for a more in-depth examination of convergent and construct validity. Finally, further investigations of how to incorporate an item related to competence both in the Norwegian version of the weight-neutral WBIS-M and other scales measuring WBI are still warranted, along with investigations on competence-related WBI across weight categories. This could include qualitative investigations of the concept of competence and WBI in focus groups.

## Conclusion

This study aimed to validate the Norwegian translation of the WBIS-M in a sample comprised of individuals of varying self-reported weight categories. Our results indicate that the Norwegian translation of the WBIS-M demonstrates satisfactory construct validity, as shown in high factor loadings and internal consistency, with a 10-item, one-factor solution consistent with previous results using the WBIS and WBIS-M. This means that the new Norwegian translation of the WBIS-M can measure WBI in Norwegian-speaking subjects of varying weights. As the first item in the WBIS pertaining to competence has been challenging in other studies [10, 11] and showed problems of possible ambiguity in our data, two versions of this item were tested, neither of which showed good fit in our CFA. The exclusion of the competence item due to psychometric concerns may be indicative of challenges with language, as the original WBIS-M did not have to exclude this item [12]. There may also be weight-dependent or cultural differences in the interpretation of the item that our sample did not allow us to explore further. However, previous concerns around the item in the WBIS points to possible challenges with this item beyond translation. It may be that the competence item is more challenging to express in a weight-neutral fashion than the remaining ten items, and future studies could examine how competence is related to WBI and its measurement qualitatively, as competence is a central concept in both weight stigma and internalized weight stigma [1, 9].

The results also showed a gender difference in WBI in our sample, with higher WBI in women than men, despite there being no significant differences in self-perceived weight, overall health, or psychological state. Furthermore, there seemed to be a more weight-independent relationship between WBI, psychological state, and overall health for women than for men. Finally, for both genders, higher WBI was related to more psychological distress, independent of self-perceived weight, underscoring the fact that WBI should be given attention in clinical settings, across weight categories and gender.

## Data Availability

The datasets generated and/or analyzed during the current study are not publicly available before the end of the project in December 2024 but are available from the last author (Fiskum) upon reasonable request. After the conclusion of the project, the data will be made available through a restricted data repository from the Norwegian government (Norwegian Centre for Research Data).

## Abbreviations

BMI: Body Mass Index
WBI: Weight Bias Internalization
WBIS: Weight Bias Internalization Scale
WBIS-M: Weight Bias Internalization Scale Modified
